# Women of ASHE: a conversation with Kristy Weinshel about the behind-the-scenes work at SHEA and leading by “The Golden Rule”

**DOI:** 10.1017/ash.2025.10073

**Published:** 2025-09-11

**Authors:** Kristy Weinshel

**Affiliations:** Society for Healthcare Epidemiology of America (SHEA), Arlington, VA, USA

## How did you become Executive Director of SHEA? What career decisions brought you here, and what drew you to SHEA’s mission?

KW: Before joining SHEA, I worked at a utilities telecom association and discovered that I loved association management. It was engaging and fulfilling. After completing my MBA, I started looking for opportunities where the mission had a more meaningful impact. Utilities just didn’t resonate with me personally. SHEA’s focus on infection prevention and antimicrobial stewardship immediately clicked. Around the time I was preparing to interview for the Executive Director role, my father-in-law passed away, and I’d seen firsthand how infection prevention impacts patient care—through him, my grandmother, and other loved ones who’ve been hospitalized. It really deepened my appreciation for this work. Everyone, at some point, is a patient or caregiver. Supporting the professionals who protect patients through infection prevention and stewardship makes this role feel mission-driven and deeply aligned with who I want to be as a leader. I truly love working for SHEA—the mission, the people, and the amazing volunteers who teach me something new every day.

## You’re very well-liked and considered highly effective by your team and SHEA members. What’s your leadership philosophy and the secret to your success?

KW: That question was so meaningful—it really filled my bucket. At the core of my leadership philosophy is the Golden Rule: treat others the way you want to be treated. I try to lead with trust, respect, and empathy, recognizing that we’re all capable humans who are constantly learning. I work to create an environment where people feel empowered, supported, and safe to take risks, recover from mistakes, and push SHEA’s mission forward. I also deeply value transparency and consistent communication. Even a small comment or discussion can spark an innovative idea down the road.SHEA is a small team, and I’m constantly amazed by how much we accomplish. That dedication inspires me to lead and also step aside to let others lead in their own ways.

## What does a typical workday look like for you and your staff in 2025, especially with healthcare being so impacted by the political landscape? What behind-the-scenes work might members not see?

KW: There’s no such thing as a typical day at SHEA—especially in 2025, with so many policy dynamics at play. What keeps us energized is the passion of SHEA members. We constantly receive ideas, concerns, and out-of-the-box suggestions from members via email or phone—and we love it. It’s our job to channel that energy into action aligned with SHEA’s mission. For example, with ACIP (Advisory Committee on Immunization Practices), we’ve worked closely with our representatives to draft public statements, coordinate media outreach, and submit formal comments—all to reflect our members’ views. These behind-the-scenes efforts—media messaging, advocacy, strategic responses—aren’t always visible, but they reflect our deep commitment to being thoughtful and proactive on behalf of the membership.

## You helped launch ASHE in 2021. What inspired the journal’s creation, and what has its growth meant to you?

KW: ASHE was a collaborative effort that I’m incredibly proud of. It wouldn’t have been possible without Lindsay MacMurray, our Director of Publications, Marketing and Membership, and our publishing partner, Cambridge University Press. We also had a dedicated task force led by volunteers like Chetan Jinadatha, MD, MPH who saw the need for a publishing space focused on stewardship. One of the most important decisions was selecting the right founding editor—Dr. Gonzalo Bearman. His credibility, energy, and connections helped attract high-quality submissions to a brand-new journal. Under his leadership, ASHE has exceeded expectations—not just as a publishing platform, but also as a revenue source and a leader in open-access publishing. Watching ASHE grow into a member-driven success story has been one of the most rewarding parts of my time as Executive Director.

## You also helped launch the popular “Women in SHEA” session, which inspired this series, “Women in ASHE”. What do you make of its success, and what topics should it tackle next?

KW: The success of “Women in SHEA” reflects so many people’s efforts. As a woman leader working with an all-female staff, I understand the unique challenges women face in trying to “have it all.” This program creates a supportive community that empowers women as they step into leadership. One moment that stayed with me was seeing the drop in female-led research during the pandemic. It reminded me why this work is so important. I’m also raising a son, and watching the rise in anti-female sentiment online in 2025 has made me even more committed to promoting equity. While the program focuses on women, I’d love to see it expand to elevate other underrepresented voices within SHEA. Our field should reflect that infection prevention and stewardship are for everyone—regardless of background or identity.

## You speak very joyfully about your son and his crazy teenage antics. What do you wish he knew about his mom as an accomplished female leader? What do you hope to teach him?

KW: Last night, my son finished his sophomore year and played with his high school lacrosse team in the state semi-finals. He didn’t get any field time except during warmups, and his team didn’t advance. He was heartbroken—not for himself, but for the seniors.He’s been practicing six days a week since February and didn’t care that he didn’t play. He just cared about the team. That moment taught me a powerful lesson: you don’t need to be a star to make a meaningful contribution. Leadership is about showing up, supporting others, and caring about collective success. I’m incredibly proud of him and humbled by what I learned from him. As much as I hope he appreciates what I do, I’m even more grateful for what he teaches me.

## Finally, what books, shows, or podcasts are you consuming—either professionally or for unwinding at the end of a long week?

Kristy Weinshel: I love documentaries—on any topic! Thankfully, a lot of the SHEA staff members do too, so we often compare notes, which helps us connect beyond just work.I also love reading. I mix leadership books with murder mysteries. Right now, I’m starting Listen for the Lie, a recommendation from Lindsay. Because we mostly work remotely, sharing what we’re reading or watching helps keep our team connected and our relationships strong.

